# The impact of built environment on physical activity and subjective well-being of urban residents: A study of core cities in the Yangtze River Delta survey

**DOI:** 10.3389/fpsyg.2022.1050486

**Published:** 2022-12-08

**Authors:** Jing Zhang, Yan Zheng, Tao Wen, Min Yang, Qiang ming Feng

**Affiliations:** ^1^Faculty of Physical Education, Shanghai International Studies University, Shanghai, China; ^2^Office of Campus Security, Shanghai International Studies University, Shanghai, China

**Keywords:** built environment, physical activities, subjective well-being, mental health, cities of Yangtze River Delta

## Abstract

**Objective:**

In cities with high population density in China, the impact of built environment on human health is rather complicated. Physical activities are an important factor in promoting people’s health. This study is aimed to explore ways of enhancing the residents’ intensity of physical activities and psychological health in a limited built environment. For this purpose, this study conducted research on 1875 residents from cities in the Yangtze River Delta in China to clarify the complicated correlations among the residents’ physical activities, the multi-dimensional geographic environment characteristics, and subjective well-being.

**Methods:**

First, Neighborhood Environment Walkability Scale (NEWS-A), International Physical Activity Questionnaire Short Form (IPAQ-SF), and Subjective Well-being Scale for Chinese Citizens (SWBS-CC) were used to measure built environment characteristics, intensity of physical activities, and subjective well-being. Second, the correlations among built environment, physical activities, and subjective well-being were analyzed, which reflected different effects of built environment characteristics on physical activities and subjective well-being. Third, physical activities were viewed as a mediating variable in SEM to analyze the influence mechanism of each built environment characteristic on the subjective well-being of residents.

**Result:**

Residents with different individual characteristics may have different levels of perception and usage of built environment. The intensity of physical activities has significant positive correlations with proximity to supporting facilities, accessibility of destinations, and public security, while no significant correlation with overall environmental aesthetics and street connectivity. The residents’ subjective well-being has significant positive correlations with accessibility of destinations, overall environmental aesthetics, and public security, while no significant correlation with proximity to supporting facilities and street connectivity. Physical activities not only have a direct effect on subjective well-being, but also a mediating effect on the correlations between subjective well-being and built environment characteristics.

**Conclusion:**

In the future, more research could be conducted on the optimization of correlations between residential built environment characteristics and physical activities as well as subjective well-being, so as to gain a deeper understanding about the impact of residential built environment on people’s physical and mental health.

## Introduction

As urbanization expands globally, cities have markedly improved people’s health with better medical services and mature infrastructure. However, the fast-speed expansion has also led to problems such as chronic diseases and mental illnesses. A large number of studies have shown that public health is closely related to built environmental factors such as urban land use, development density and transportation system (Lin and Yang, 2015). A good residential environment can not only promote healthy physical activity, reduce the risk of obesity, cardiovascular disease and other chronic diseases, but also provide people with psychological recovery and mental release, thus promoting mental health and improving happiness (Zhao et al., 2022). In 1984, WHO initiated the Healthy Cities Campaign and identified the urban built environment as an important factor affecting public health. Thus, the built environment became an important entry point for human settlements planning to actively intervene in health (Lu and Tan, 2015).

Social ecological model, the theoretical basis of healthy city, believes that people’s health status is subject not only to their genes and roles in social and economic lives, but also to the external built environment, social environment, and natural environment (Sallis, 2009). As technologies such as GIS, RS, and GPS get mature, scholars of geographics start to consider built environment characteristics and believe that a positive and friendly urban environment encourages the residents’ participation in physical activities and is conducive to human health (Lu and Qin, 2013).

Built environment refers to the sum of physical characteristics that encompass the surroundings, or a spatial combination of various physical environmental characteristics such as land use, transportation system, and infrastructure (Timmermans et al., 2016). In early time, Cervero et al. proposed a 3D conceptual model of built environment, which comprises density, diversity, and urban design (Cervero and Kockelman, 1997). On that basis, Ewing et al. added two more concepts, namely accessibility of destinations and distance to transit, forming a new “5D” model (Ewing and Cervero, 2001). In research on built environment and physical activities by Boarnet and others, environment characteristics such as population density, employment density, distance to transit, and distance to city center exhibit a significant impact on residents’ physical activities (Boarnet et al., 2008). The higher the degree of mixed land use, the more convenient it is for people to travel. When the travel distance gets shorter, people are more likely to choose green travel and thus have more physical activities (Saelens and Handy, 2008). Oakes et al. also found that, a compact community of high density could encourage the residents to travel more on foot or by bike, which also means more physical activities (Oakes et al., 2007; Forsyth et al., 2008). Besides, the distance from the residential area to the entertainment area, well-equipped sports courts, well-designed streets, and walkable residential environment also have significant correlations with green travel frequency and physical activities (Koohsari et al., 2015). According to existing literature, the academia has reached a consensus on how destination accessibility and distance influence the residents’ physical activities: the higher the accessibility of destinations, the shorter the travel distance, the more likely that people would travel on foot or by bike, and the more physical activities they will have (Heinen et al., 2009). According to Kerr, Galvez, Wendel et al., people’s walking is positively correlated to sidewalks connection. A clearer street network helps increase physical activities like walking or biking (Kerr et al., 2006; Wendel-vos et al., 2007; Galvez et al., 2009). Children and teenagers would participate more in outdoor activities when they believe there is a safe and large activity space (Nuno et al., 2010).

Mental health is usually reflected in two aspects, namely positive development and no psychological disorders (Robert, 2012). Positive development means realizing subjective well-being, perceived self-efficacy, autonomy, intelligence, and emotional potentials (World Health Organization, 2001). Subjective well-being is often known as a person’s subjective feelings affected by his or her possession of environmental resources during his or her interactions with the external environment and is closely related with subjective behaviors (Pacione, 2003). It could be influenced by various factors, which can be summarized into two aspects: external environment characteristics (e.g., behaviors, social culture, education, economy, geography, life incidents, aesthetics) and individual characteristics (e.g., genetics, cognition, personalities, moral ethics; Steptoe, 2019). Good built environment design can guide the living state of residents and thus affect the subjective well-being. In research on built environment and subjective well-being by Dong and others, a higher degree of mixed land use improves living convenience and thus people’s well-being (Dong et al., 2018). Ma and Rao believed that a higher land-use mixing degree may be beneficial to improve residents’ happiness due to its convenience (Ma et al., 2020). Neil Harris and Huw Thomas also found that diversified public activity places and perfect entertainment facilities can improve the quality of life and happiness of residents (Harris et al., 2008). Su and Zhou believed that the increase in the number of daily living facilities can improve residents’ subjective well-being (Su et al., 2019). But other studies have found no significant relationship between diversity and happiness (Cao, 2016; Jie and Bindong, 2017). While the correlations between subjective well-being and accessibility of destinations, including accessibility to medical facilities, entertainment, accessible stores, and education institutions, vary in different research regions and groups. However, most existing results show positive relationships between subjective well-being and accessibility of destinations (Brereton et al., 2011; Morrison, 2011). Ambrey and Fleming believed that in the design of urban built environment, green landscape also has a positive effect on people’s mental health and well-being (Ambrey and Fleming, 2014). According to Ballas and Tranmer, a good security situation can often improve the subjective well-being of individuals (Ballas and Tranmer, 2012).

From the perspective of behavior studies, human sense of well-being could result from various behaviors for various reasons. In addition to directly affecting people’s physical activity and subjective well-being, built environment can also have indirect effects on subjective well-being through sports participation behavior. From the perspective of “social psychology,” people’s psychological perception should include happiness, which is the unique experiences going through by individuals (Taylor et al., 1997). Sports participation can eliminate mental tension and stress, reduce depression, enhance physical fitness, and thus produce subjective well-being (Donaldson et al., 2011). From the perspective of “social capital,” through social interaction and social communication with others in the process of sports participation, individuals can gain social capital like trust or social support (Frey and Stutzer, 2010), which in turn could escalate individual’s happiness level (Krause and Wulff, 2005; Dolfsma and Davis, 2009; Growiec and Growiec, 2011). Most of the empirical results also show that sports participation has a certain positive effect on subjective well-being (Liu, 2016; Schmiedeberg and Schröder, 2017). In Craveiro and Erin’s research, they pointed out that accessibility to public space like green land and parks not only directly promotes human health but also indirectly by creating opportunities for outdoor physical activities, and increasing social interactions and community participation (Erin et al., 2015; Craveiro, 2017). Florindo studied the relationship between leisure-time walking and the presence of public open spaces such as parks, bike paths and squares, and showed positive correlation between promotion of physical activity among adults and the existence of aforementioned open spaces (Florindo et al., 2017). However, some other scholars found that such effects on health were overestimated. For example, Kawachi and Berkman thought that health-promotion environment may not bring much improvement in public health, while collective community operation, which includes culture and norms, community integration, community support system, etc. turns out to be a more decisive social factor for individual health (Kawachi and Berkman, 2000; Lindstrom et al., 2002).

Urbanization, fast growing population, gathering of industries in big cities have caused huge demands for all type of resources and have led to a series of environmental issues, and they are becoming a great impact on living environments of human being as well as threatening human being’s safety and health. As design for healthy living has become a new trend in developed countries, it is important to emphasis public health as a crucial option in urban residential built environment design and construction (Patrick et al., 2005). Although many scholars from highly urbanized countries have found significant correlations between built environment, physical activities, and subjective well-being in their research. However, the impact mechanism of the built environment on people’s physical and mental health is very complex, due to comprehensive impact of cultural, social and economic conditions and personal lifestyle. On the one hand, for the action of built environment on physical activity and subjective well-being, the impact mechanism is still weak, and the impact path is still not clear enough. On the other hand, these results cannot be directly referenced from cities with high population density in developed countries as most of them are in Europe and the US where there is sparse population and advanced economy. The Yangtze River Delta region is the area with the highest degree of urbanization, the densest urban distribution and the fastest population growth in China. It is also the area with rapid growth of urban expansion. The population growth rate is fast and the density is relatively concentrated. The convergence of population, land, and capital in cities had boosted the continuous outward expansion of urban (Huang et al., 2021). Therefore, it is of great significance to study how to build urban residential built environment of the cities in the Yangtze River Delta of China to promote public health during unban expansion. The results could be referenced for other rapidly expanding cities especially cities in those developing countries with similar urban expansion scale as in the Yangtze River Delta of China.

**Figure 1:**
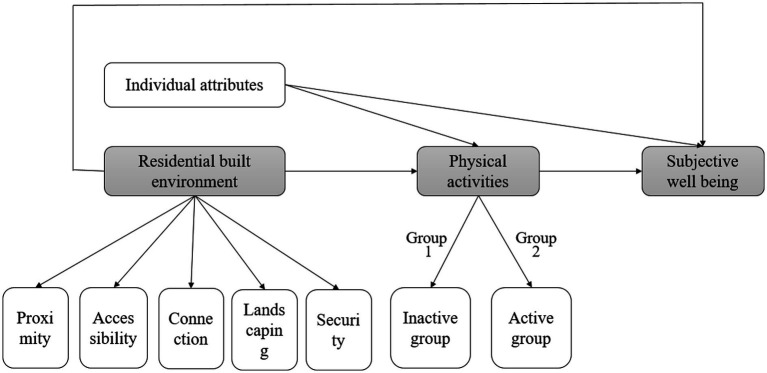
Theoretical framework of “Environment-Activity-Health.”

This study, based on the existing research results in domestic and foreign academia and by using the micro research paradigm of individual behaviors, proposed a theoretical framework featuring “built environment – physical activity – subjective well-being” after investigating the complicated correlations among the residents’ daily behaviors, the multi-dimensional geographic environment characteristics, and subjective well-being. In this analysis model, individual characteristics are the controlled variables, built environment characteristics are the independent variables, and physical activities and subjective well-being are the dependent variables (Figure 1). Based on the analysis above, three hypotheses are put forward as follows:

*Hypothesis 1*: Built environment characteristics have a positive effect on the residents’ physical activities.

*Hypothesis 2*: Built environment characteristics have a positive effect on the residents’ subjective well-being.

*Hypothesis 3*: Physical activities have a mediating effect on the correlations between subjective well-being and built environment characteristics.

## Materials and methods

### Study area

This study selects the expanding cities in the Yangtze River Delta as the study area. Hereby the expanding cities mainly refer to mega cities, super-large cities, and large cities with urban population over 1 million (Fang, 2014), and they have the potential to have a growing population in the near future. According to the Yangtze River Delta Urban Agglomeration Development Plan approved by the State Council in 2016, the Yangtze River Delta will be built into a world-class urban agglomeration of world-class quality by 2030. In the future, regional population and economic factors will further gather, urban space will further expand, and the environmental impact of urbanization will further intensify. Shanghai, Hangzhou, Nanjing and Hefei, as important cities in the Yangtze River Delta urban agglomeration, have witnessed rapid growth in urbanization rate in recent years. As of 2020, the urbanization rate of Shanghai has reached 89.3%, and the urbanization rate of the other four cities have also been above 80%.

### Study design and participants

This study used a cross-sectional survey on the relationship between the environment characteristics in the residential areas in cities of Yangtze River Delta and the residents’ physical activities and subjective well-being. Research subjects are urban residents in Shanghai, Hangzhou, Nanjing, Hefei, whose residential areas all present multiple built environment characteristics. This study ran from February to August 2022. Choose different types of communities in each city, such as traditional old communities and modern commercial housing communities. A total of 2,500 questionnaires were randomly distributed through the neighborhood committees of each community. 2,037 samples were recovered with a recovery rate of 81.48%. Respondents aged over 65 were instructed by trained interviewers when filling in the questionnaires. After deleting the invalid answers, the study collected 1875 questionnaires (the effective rate is 91.95%), among which 893 were from male respondents and 982 were from female respondents.

And this study complies with the Declaration and Helsinki, and ethical approval was granted by the Chinese ethics research review board. All respondents agreed to the experimental agreement and signed an informed consent form.

### Measures

#### Measure of residential built environment characteristics

Residential built environment characteristics were measured with the abbreviated form of Neighborhood Environment Walkability Scale (NEWS-A) by Cerin et al. to evaluate the respondents’ perception of their residential areas related to physical activities (Cerin et al., 2006). The scale has been verified to be effective when used in Chinese Mainland (Zhou et al., 2011; Yukun et al., 2012). It includes 24 items in 5 dimensions, namely proximity to supporting facilities, accessibility of destinations, street connectivity, overall environmental aesthetics, and public security. Proximity to supporting facilities means whether there are outdoor courts, indoor sports venues, sidewalks or cycling roads nearby and whether the sports equipment is available, 5 questions in total. Accessibility of destinations refers to the accessibility from the residential area to public areas like sports venues, squares, bus stations, shopping malls, and parks, 6 questions in total. Street connectivity refers to the number of crossroads and streets, and the conditions of street lighting, pavement, and hygiene, which reflect the convenience of accessing public areas like sports venues, shopping malls, and parks, 4 questions in total. Overall environmental aesthetics is the aesthetic attraction of the surrounding natural environment, buildings, greenness, and public green sidewalks, 5 questions in total. And public security is the security level of the residential area and its traffic conditions, 5 questions in total. The scale is designed in five-point style, where 1 stands for “totally disagree,” 2 for “disagree a little,” 3 for “neutral,” 4 for “agree a little,” and 5 for “totally agree.” The score of each dimension is the average score of all items in each dimension. Higher points indicate higher degrees of satisfaction toward residential built environment (Xie and Peng, 2021). In this measurement: Cronbach’s α = 0.953, KMO = 0.910, and Bartlett *p* < 0.001. Internal consistency reliability for the 5 dimensions: respectively 0.942, 0.832, 0.868, 0.857, and 0.873, indicating that the scale is of well validity and reliability and is suitable for factor analysis.

#### Measure of physical activities

The measure of physical activities in this study adopted the International Physical Activity Questionnaire Short Form (IPAQ-SF), which is commonly used in Chinese Mainland and is regarded as a scientific tool for measuring adults’ physical activities (Qu and Li, 2004). The questionnaire is composed of seven questions and categorizes activity intensity into three levels, namely low, medium, and high levels (Elahi et al., 2021, 2022). According to the frequency and time of three kinds of physical activities per week, the physical activities level of residents can be calculated and the physical activities participation of residents can be measured. The specific formula is:

Physical activities level = (3.3 × A × a) + (4 × B × b) + (8 × C × c).

A, B and C represent the frequency of low, medium and high intensity physical activities. a, b and c represent the average time spent doing low, medium and high intensity physical activities. 3.3, 4, and 8 represent the metabolic equivalent (METs) assignment of each low, medium and high intensity physical activities (Jing and Chunzheng, 2021).

Evaluation standards for different intensity levels of weekly physical activities are shown in Table 1. Research subjects are further divided into two groups, namely the active group and the inactive group based on their intensity of physical activities. The active group include those of high-and medium-level physical activities and the inactive group include those of low-level physical activities.

**Table 1:** Evaluation standards for different intensity levels of weekly physical activities (Chen et al., 2019).

Intensity levels of physical activities	Evaluation Standards
High-level physical activities (meeting any of the two standards)	(1) Over 3 days of high-level physical activities a week, with 1,500-MET energy consumed in total.
	(2) Seven days of high-or medium-level physical activities a week, with 3,000-MET energy consumed in total.
Medium-level physical activities (meeting any of the three standards)	(3) At least 3 days of high-level physical activities a week, each over 20 min.
	(4) At least 5 days of medium-level physical activities, or walking for over 30 min every day.
	(5) At least 5 days of high-or medium-level physical activities a week, with 600-MET energy consumed in total.
Low-level physical activities	(6) Not meeting the standards of high-or medium-level physical activities.

#### Measure of subjective well-being

The subjective well-being was measured with the Subjective Well-being Scale for Chinese Citizens (SWBS-CC20) by scholar Xing Zhanjun from Chinese Mainland. With reliability of 0.848 and validity of 0.972, the scale is an effective measurement in research of urban citizens in Chinese Mainland. This scale is a six-level Likert scale. It contains 20 items in 10 dimensions (Each dimension contains two items), including satisfaction and abundance, mental health, confidence towards society, growth and progress, goal and personal value, self-acceptance, physical health, psychological balance, adaptation to interpersonal relations, and family atmosphere (Xin, 2003). Each question in the subjective well-being scale form used in this study adopts a 6-level scoring system, which is divided into “completely disagreed, disagreed, slightly disagreed, slightly agreed, agreed and completely agreed,” with 1 to 6 points, respectively. Question number 4, 5, 6, 9, 10, 11, 13, 15, 17, 18, and 20 are scored inversely. The overall well-being score is the average of the total scores of 20 questions. According to Xing, the higher the respondent’s score, the happier he is. A score above 4.5 is considered high, and a score below 2.5 is considered low (Xin, 2009). In this measurement: Cronbach’s α = 0.856, KMO = 0.879, and Bartlett *p* < 0.001, indicating that the scale is of well validity and reliability and is suitable for factor analysis.

### Statistical analyses

The statistical analyses were conducted with SPSS 23.0 and Amose23.0. The level of statistical significance was set at *p* < 0.05. The descriptive characteristics were summarized and presented as mean and standard deviation (SD), count and percentages. Firstly, this study used SPSS to conduct descriptive statistics on the differences in the perception of the built environment, physical activity and subjective well-being of residents with different individual characteristics.

Secondly, people may have different levels of perception and usage of built environment due to different individual characteristics (Smith et al., 2012; Wang and Jiao, 2018). Therefore, these individual characteristics were measured at baseline and were incorporated as potential confounders in the regression model. This study first conducted collinearity diagnostics on the variables of individual characteristics (gender, age, educational attainment, income) and built environment (proximity to supporting facilities, accessibility of destinations, street connectivity, overall environmental aesthetics, public security). According to the results, there is no collinearity between built environment and individual characteristics (Sun, 2005). Then, independent sample t-test and one-way ANOVA were used to select controlled variables through the test of the difference between physical activity, subjective well-being and gender, age, educational attainment, and income.

Thirdly, to verify the correlations between physical activities and residential built environment, independent sample t-test was used to compare the perception of built environment of respondents of different intensity levels of physical activities. With variables controlled, the correlations between physical activities and proximity to supporting facilities, accessibility of destinations, street connectivity, overall environmental aesthetics, and public security were tested with binary logistic regression. And to verify the correlations between subjective well-being and residential built environment, with variables controlled, the correlations between subjective well-being and proximity to supporting facilities, accessibility of destinations, street connectivity, overall environmental aesthetics, and public security were tested with linear regression. The results were presented as OR with 95% confidence interval (CI).

Finally, and the study adopted Bootstrap (under 95% CI and sampling for 5,000 times) to figure out whether the physical activities have a mediating effect on the correlations between subjective well-being and residential built environment.

## Results

### Descriptive analysis results of the variables

Dependent variables are the residents’ physical activities and subjective well-being. Independent variables include the 5 dimensions of built environment, namely proximity to supporting facilities, accessibility of destinations, street connectivity, overall environmental aesthetics, and public security. Individual attributes variable refer to Demographic variables, including gender, age, educational attainment, and income. The descriptive analyses were conducted on the physical activities, subjective well-being, and residential built environment characteristics.

As shown in the results, the inactive group has 765 respondents, accounting for 40.80% of the total and the active group has 1,110, accounting for 59.20% of the total. The average score of well-being reached 4.16, which is a relatively high level. Among the perceived scores of the five dimensions of the built environment, public security’s > overall environmental aesthetics > accessibility of destination > proximity to supporting facilities > street connectivity (Table 2).

**Table 2:** Results of descriptive analyses of the variables.

Variable		Total (*n* = 1875)	M (%)	SD
Dependent variable	**Physical activities**	1875		
	Inactive group	765	40.80	
	Active group	1,110	59.20	
	**Subjective well-being**	1875	4.16	0.63
Independent variable	**Built environment**	1875		
	Proximity to supporting facilities	1875	3.57	0.89
	Accessibility of destinations	1875	3.59	0.94
	Street connectivity	1875	3.54	1.23
	Overall environmental aesthetics	1875	3.60	0.82
	Public security	1875	3.85	0.81
Individual attributes variable	**Gender**			
	Male	893	47.60	
	Female	982	52.40	
	**Age**			
	14 < 25	457	24.40	
	25–45	468	25.00	
	46–59	576	30.70	
	≥60	374	19.90	
	**Educational attainment**			
	High school or less	658	35.10	
	Vocational college	343	18.30	
	Undergraduate degree	673	35.90	
	Graduate degree or above	201	10.70	
	**Income**			
	<4,000 RMB	444	23.70	
	4,000–8,000 RMB	1,054	56.20	
	>8,000 RMB	377	20.10	

### Perception differences of built environment, physical activity, and subject well-being by residents with different individual characteristics

#### Difference analysis on perception of built environment By residents with different individual characteristics

Firstly, as found in the survey on the perception of built environment conducted on residents with different individual characteristics (Table 3): female residents share the similar scores in four out of five perceptional assessments, except in perception of public safety, where female scored (3.80) slightly lower than male (3.91). For residents age 14–25, they scored the highest, whereas for residents age between 25 and 45, they scored the lowest. Furthermore, for residents with varying educational background, those with high school diploma or below scored the highest. For residents with different financial status, those with middle or high incomes scored higher in general than those with lower incomes.

**Table 3:** Perception differences of built environment, physical activity, and subject well-being by residents with different individual characteristics.

Variable	Proximity to supporting facilities	Accessibility of destinations	Street connectivity	Overall environmental aesthetics	Public security	Inactive rate	Subjective well-being
**Gender [M (%)]**							
Male	3.55	3.59	3.50	3.57	3.91	43.73	4.12
Female	3.59	3.60	3.54	3.61	3.80	39.93	4.19
**Age [M (%)]**							
14 < 25	3.77	3.88	3.76	3.73	3.97	16.38	4.21
25–45	3.39	3.50	3.28	3.49	3.71	50.18	4.00
46–59	3.58	3.57	3.54	3.61	3.88	40.46	4.30
≥60	3.55	3.40	3.49	3.55	3.85	61.72	4.10
**Educational attainment [M (%)]**							
High school or less	3.70	3.71	3.64	3.75	4.00	41.83	4.23
Vocational college	3.52	3.28	3.27	3.50	3.73	52.91	4.08
Undergraduate degree	3.48	3.64	3.56	3.53	3.83	35.51	4.07
Graduate degree or above	3.56	3.62	3.41	3.48	3.70	40.65	4.26
**Income [M (%)]**							
<4,000 RMB	3.55	3.58	3.39	3.59	3.80	46.67	4.02
4,000–8,000 RMB	3.60	3.55	3.56	3.61	3.87	40.48	4.16
>8,000 RMB	3.54	3.73	3.57	3.55	3.88	37.19	4.29

#### Difference analysis on physical activity of residents with different individual characteristics

Secondly, as found in the survey regarding physical activities of residents with varying individual characteristics, inactivity is more common among females than males. The study also shows that among residents age 14–25, the inactive rate is the lowest (16.38%), among residents age between 46 and 59, the rate is 40.46%, and among senior residents, the rate is 61.72%. Moreover, the results vary among residents with different educational background: residents with undergraduate degree have the inactive rate of 35.51%, compared to the inactive rate of 52.91% among residents with vocational college degree, and 40.65% with graduate degree or above. The results also suggest that inactivity vary among residents with different financial status. Those with high incomes have the lowest inactive rate (37.19%), those with medium incomes have the rate of 40.48%, and those with low incomes have the highest rate of 46.67%.

#### Difference analysis on subject well-being of residents with different individual characteristics

Finally, as found in the survey on the subjective well-being of residents with different individual characteristics: women’s subjective well-being (4.19) is higher than men’s (4.12). For residents of different ages, people aged 46 to 59 have the greatest sense of subjective well-being (4.30). However, people aged 25–45 (4.00) and over 60 (4.10) had relatively low subjective well-being. For residents with different levels of educational attainment, the highest score goes to graduate degrees above (4.26). And for residents of different economic statuses, their subjective well-being decreases successively from the high-income group (4.29) to the low-income (4.02).

#### The impact of the variables of residential built environment on the residents’ physical activities and subjective well-being

With a normal distribution of the data, this study further verified the correlations between the residents’ physical activities, subjective well-being, and built environment characteristics. According to the results, every two variables are positively correlated with *p* < 0.01, which is of statistical significance. Besides, the results indicate independence of the variables, meaning they are valid for further statistical analyses.

#### The impact of the variables of residential built environment on the residents’ physical activities

Independent sample t-test was used to compare the scores of respondents of different intensity levels of physical activities (the active group and the inactive group). As shown in Table 4, the active group has higher average scores in proximity to supporting facilities, accessibility of destinations, street connectivity, overall environmental aesthetics, and public security than those of the inactive group. All the data in the table have significant difference (*p* < 0.01).

**Table 4:** Correlation analysis of physical activities and residential built environment characteristics.

Variable	Inactive group (*n* = 765)	Active group (*n* = 1,110)	*t*	*p*
	M SD	M SD		
Proximity to supporting facilities	3.295 ± 0.900	3.765 ± 0.834	−11.593	0.000
Accessibility of destinations	3.102 ± 0.947	3.933 ± 0.779	−20.791	0.00
Street connectivity	3.222 ± 1.252	3.762 ± 1.182	−9.487	0.00
Overall environmental aesthetics	3.407 ± 0.882	3.729 ± 0.745	−8.534	0.00
Public security	3.633 ± 0.794	4.009 ± 0.800	−10.040	0.00

Binary logistic regression was used to analyze the correlations between the residents’ physical activities and residential built environment characteristics. Based on above, analyses were conducted with age as the controlled variable, physical activities as the dependent variable, and the five residential built environment characteristics as the independent variables. As the results shown in Table 5, three out of the five environment characteristics, namely proximity to supporting facilities, accessibility of destinations, and public security are of statistical correlations with physical activities, or in other words, have impact on physical activities. More specifically, the residents would have more physical activities when the community is of high security level and has supporting facilities and sports venues that are easy to access. While no significant correlation was discovered between physical activities and street connectivity as well as overall environmental aesthetics.

**Table 5:** Logistic regression analysis of physical activities and residential built environment characteristics.

Variable	*B*	OR	*p*	95%CI	
				Lower	Upper
(constant)	−4.583	0.010	0.000		
Proximity to supporting facilities	0.139	1.149	0.030	1.013	1.303
Accessibility of destinations	1.021	2.775	0.000	2.410	3.194
Street connectivity	0.051	1.052	0.298	0.956	1.158
Overall environmental aesthetics	0.132	0.876	0.140	0.735	1.044
Public security	0.339	1.404	0.000	1.193	1.652

#### The impact of the variables of residential built environment on the residents’ subjective well-being

In the analysis of the correlations between the residents’ subjective well-being and residential built environment characteristics, linear regression was adopted. Based on above, analyses were conducted with age, educational attainment, and income as the controlled variables, subjective well-being as the dependent variable, and the five residential built environment characteristics as the independent variables. The results are shown in Table 6, which presents statistical correlations between subjective well-being and three environment characteristics, namely accessibility of destinations, overall environmental aesthetics, and public security. In other words, residents’ subjective well-being could be influenced by these three characteristics. People would feel happier when there are easy-to-access sports courts, a beautiful environment, and reliable public security. While the rest two environment characteristics, namely proximity to supporting facilities and street connectivity, were found to have no significant correlations with subjective well-being.

**Table 6:** Regression analysis of subjective well-being and residential built environment characteristics.

Variable	*B*	Beta	*t*	*p*	95%CI	
					Lower	Upper
(constant)	3.066	0	30.631	0.000	2.870	3.262
Proximity to supporting facilities	0.008	0.012	0.393	0.694	−0.033	0.050
Accessibility of destinations	0.040	0.060	2.489	0.013	0.009	0.072
Street connectivity	−0.017	−0.034	−1.369	0.171	−0.042	0.007
Overall environmental aesthetics	0.091	0.117	4.062	0.000	0.047	0.134
Public security	0.174	0.022	8.028	0.000	0.132	0.217

#### Structural equation model on the correlations between residential built environment, physical activities, and subjective well-being

**Figure 2:**
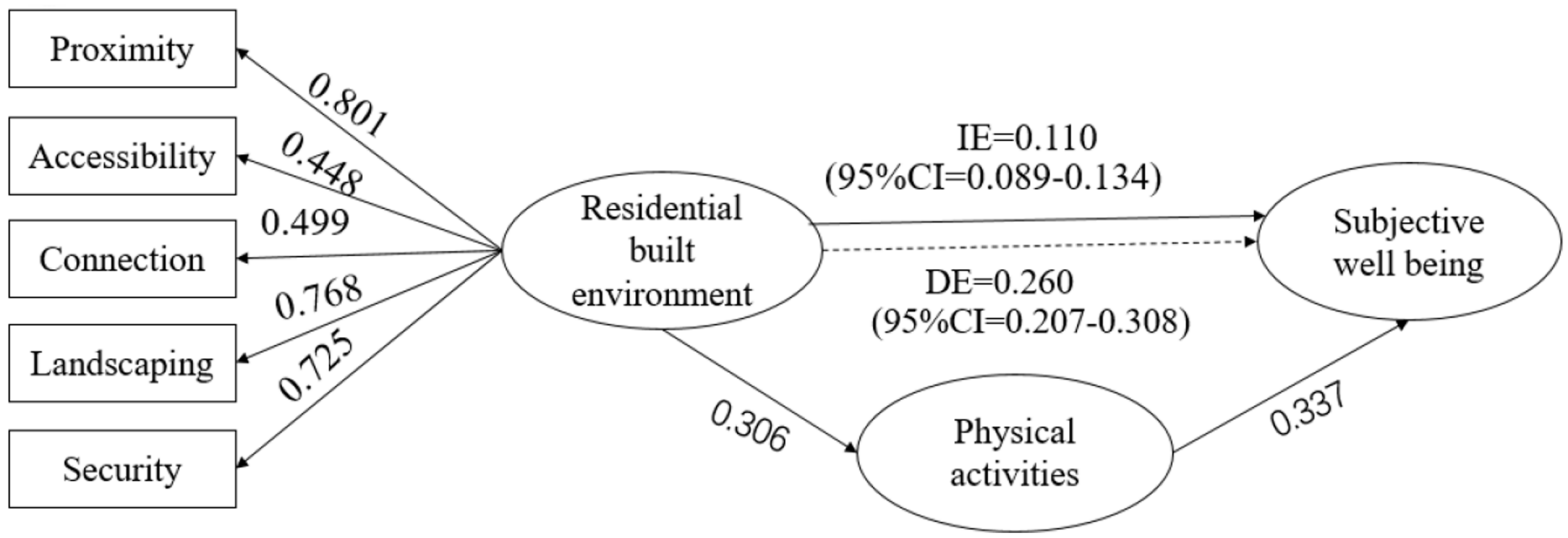
Mediator model on residential built environment, physical activities, and subjective well-being.

This study constructed a structural equation model (Figure 2) on the correlations between residential built environment, physical activities, and subjective well-being based on the factor analyses on the data collected from the 1875 questionnaires. Table 7 presents the fit indices of the model. With *χ*2/*df* = 5.183, RMSEA = 0.078, GFI (0.952)/AGFI (0.913)/NFI (0.95)/CFI (0.953)/IFI (0.953) > 0.9, and PNFI (0.543)/PCFI (0.545) > 0.5, the fit of the model is within the acceptable range.

**Table 7:** Path test of residential built environment, physical activities, and subjective well-being.

Path	Non-standard coefficient	Standard coefficient	S.E.	C.R.	P
Built environment——Physical activities	0.253	0.306	0.021	11.992	0.000
Physical activities——Subjective well-being	0.433	0.337	0.028	15.442	0.000
Built environment——Subjective well-being	0.260	0.244	0.026	9.931	0.000

From Table 7, Figure 2, it can be inferred that residential built environment has a direct effect on both people’s physical activities and subjective well-being, with a path coefficient of 0.306 and 0.244, respectively. Many past research has proved physical activities’ function in improving subjective well-being, and their correlations are verified in this study, that is, physical activities have a direct effect on subjective well-being, with a path coefficient of 0.337. Moreover, the loadings of the environment characteristics are, respectively, 0.801 for proximity to supporting facilities, 0.768 for overall environmental aesthetics, 0.725 for public security, 0.499 for street connectivity, and 0.448 for accessibility of destinations.

This study also adopted Bootstrap (under 95% CI and sampling for 5,000 times) to test whether physical activities have a mediating effect on the correlations between subjective well-being and residential built environment. As shown in Table 8, the bias-corrected intervals of physical activities’ mediating effect (0.089–0.134) and direct effect (0.207–0.308) are higher than the value of 0, indicating significant effects in both paths, respectively accounting for 29.730 and 70.270%.

**Table 8:** Total, direct, and mediating effects of physical activities.

Path	Effect value	SE	Bias-corrected 95%CI lower upper		*p*	Effect percentage (%)
Mediating effect	0.110	0.011	0.089	0.134	0.000	29.730
Direct effect	0.260	0.026	0.207	0.308	0.000	70.270
Total effect	0.370	0.026	0.317	0.419	0.000	

This study further uses the intermediary analysis method to test whether physical activity has intermediary effects in the five characteristics of the built environment, and the test is strictly in accordance with the intermediary effect test process provided by Wen and Ye (2014). C represents the total effect when independent variable X corresponds to variable Y; A represents the effect of the independent variable X on M, b represents the effect of the corresponding variable Y of M, and A *b is the mediating effect. C ‘represents the direct effect of X on Y after controlling for the mediating variable M. The conclusion of mediating effect are shown in Table 9:Physical activity played a complete mediating role in Proximity to supporting facilities and Subjective well-being. Overall environmental aesthetics and Public security not only directly affect residents’ subjective well-being, but also have indirect effects on subjective well-being through physical activities. The indirect effect value accounted for 14.73 and 16.75% of the total effect value.

**Table 9:** Mediating effect of intensity of physical activities on built environment characteristics and subjective well-being.

Path	c Total effect	a	b	a*b Mediating effect	a*b (95% boot CI)	c’ Direct effect	Conclusion
Proximity to supporting facilities = > Physical activities = > Subjective well-being	0.071**	0.205**	0.490**	0.101	0.128 ~ 0.174	0.03**	Completely Mediator
Accessibility of destinations = > Physical activities = > Subjective well-being	0.008	−0.004	0.490**	−0.002	−0.021 ~ 0.015	0.002	No significant Mediator
Street connectivity = > Physical activities = > Subjective well-being	−0.001	0.011	0.490**	0.005	−0.006 ~ 0.028	−0.007	No significant Mediator
Overall environmental aesthetics = > Physical activities = > Subjective well-being	0.076**	0.023*	0.490**	0.011	0.002 ~ 0.035	0.065**	Partial Mediator
Public security = > Physical activities = > Subjective well-being	0.122**	0.042**	0.490**	0.02	0.002 ~ 0.035	0.101**	Partial Mediator

*p < 0.05, **p < 0.01.

## Discussion

### Perception features of built environment, physical activity, and subject well-being by residents with different individual characteristics

As China is under rapid development of urbanization, people have raised more diversified and personalized demands for urban lives, which are also reflected in their varying demands towards built environment. This study has proved that residents with different individual characteristics may have different levels of perception and usage of built environment (Smith et al., 2012; Wang and Jiao, 2018). This result is consistent with Cao et al., that compared with men, women are more sensitive in perceiving the microenvironment (Gao et al., 2022). The reason may be that as a vulnerable group, women are also more sensitive to environment security. The research also shows that the young and middle-aged people (25–45 years old) are less satisfied with the overall built environment while the teenagers present the greatest satisfaction. This result is consistent with Wu et al., that it is maybe because the young and middle-aged people bear more pressure and responsibility for work and family. Also, their requirements for built environment may be higher than the teenagers’ (Wu et al., 2013). The high-income people show a relatively high level of satisfaction towards built environment and the low-income the least. Probably because people with higher income live in better communities with more convenient public transportation, public services, and facilities such as sports venues.

Individual characteristics including gender, age, educational attainment, and income all have an impact on the residents’ participation in physical activities (De and Sallis, 2002; Poggio et al., 2016). This study has proved that people with different individual characteristics have different intensities of physical activities. Most research has concluded that lack of physical activities is more likely to happen to women than to men (Vare et al., 2003), but some attributed that to a regional issue. The study of Qiao show that in some regional men are less physically active than women (Qiao and Wang, 2015). According to analyses of this study, men have fewer physical activities than women. Meanwhile, among different age groups, those between 46 to 50 years old have more physical activities than others. The reason may partially lie in retirement. Lahti et al. found that men near retirement would increase the time spent on medium-level physical activities by 42 min every week and women by 31 min (Lahti et al., 2011). In France, regardless of gender, people spend 2 more hours in physical activities every week after retirement (Touvier et al., 2010). In China, most women retire at the age of 50 and men at the age of 60. The retired people, especially women who retire much earlier than men, have more spare time for more physical activities. Individual characteristics of educational attainment and income present a non-linear pattern. But overall, people with undergraduate degrees or above tend to have higher intensity of physical activities than those less educated, which may be related to their awareness of the importance of physical activities. This result is consistent with Prochaska et al., that people with higher educational attainment are more likely to engage in mental work or jobs that require little physical strength. Although having fewer chances of commuting by walking or cycling, they would have more leisure physical activities as they know it is important to do so (Prochaska, 2011).

The residents’ individual characteristics, namely gender, age, educational attainment, and income also have a significant impact on their subjective well-being (Helliwell and Putnam, 2004; Dolan et al., 2008; Sweet and Kanaroglou, 2016). This study has proved the relevant hypothesis. This result is consistent with the study of Gerdtham, Lin and McBride et al. Compared with men, women are easier to develop a sense of well-being (Gerdtham and Johannesson, 2001). A “U” type correlation, which decreases first and increases later, was found between age and subjective well-being (Lin and Sun, 2017). People with higher educational attainment would have greater subjective well-being. And higher income could also effectively improve subjective well-being (McBride, 2001).

### The impact of residential built environment on physical activities

Factors that may influence people’s physical activities are complicated and keep changing over time. The socio-ecological model regards residential built environment and public facilities as two of these factors. For example, sufficient convenient public facilities, a beautiful environment, high-level public security, low traffic flow, etc. could encourage the residents to have more physical activities (Cranney et al., 2016; Yang et al., 2017). Therefore, residential built environment is an important influencing factor of physical activities. This study verified the significant correlations between the residents’ physical activities and the five residential built environment characteristics, namely proximity to supporting facilities, accessibility of destinations, street connectivity, overall environmental aesthetics, and public security. Also, residential built environment’s direct effect on the elderly residents was proved with structural equation model.

Besides, in this study, residents of the active group are more satisfied with factors including proximity to supporting facilities, accessibility of destinations, and public security than those of the inactive group. And it is found that people would have more physical activities when there are sufficient convenient supporting facilities, easy-to-access sports venues, and high public security level. This result is consistent with those concluded by scholars in foreign academia. For example, Jansen et al. concluded that, better public facilities, especially entertainment facilities could increase the adults’ physical activities and thus improve their health (Jansen et al., 2016). According to Van and others, safe traffic and convenient public supporting facilities that are easy to access are also conducive to the residents’ physical activities (Van et al., 2012). From the above, it can be inferred that a community with good sports facilities, venues, and public areas like parks and squares that are suitable for sports exercises, as well as high public security level and low traffic flow has a certain strength in enhancing people’s motivation for sports exercises and improving their status of physical activities.

No significant correlation was found between physical activities and the rest two built environment characteristics, namely overall environmental aesthetics and street connectivity. Though in some research, more streets and better street connectivity could encourage walking or cycling trips as the travel distance gets shorter and people have more choices of traveling routes (Helbich et al., 2016). Some scholars concluded that street connectivity is negatively related to the residents’ walking. In an environment of high street connectivity, the elderly would have a reduced sense of security towards public transport and thus spend less time on physical activities every week (Van et al., 2011). It may be because overall environmental aesthetics and street connectivity emphasize more the improvement of urban greenness and sceneries as well as traffic conditions, which are aimed either to enhance the community’s ecological and esthetical functions or to reduce its traffic jams and potential traffic danger. Providing more areas for physical activities is not the priority in the planning of overall environmental aesthetics and street connectivity. Instead, it may weaken the residents’ enthusiasm for physical activities. Hence, tests on the correlations between physical activities, overall environmental aesthetics and street connectivity could draw different results.

### The impact of residential built environment on subjective well-being

Subjective well-being receives multiple influences from both the physical environment and the subjective social environment. The influencing factors could be temporal-special behaviors or the multi-dimensional geographical environment. For example, land use mix, convenience and accessibility of public facilities, traffic connectivity, and the overall environmental aesthetics could improve residential subjective well-being (Ma et al., 2018). Therefore, residential built environment is an important factor that may influence subjective well-being. This study verified the significant correlations between the residents’ subjective well-being and the five residential built environment characteristics, namely proximity to supporting facilities, accessibility of destinations, street connectivity, overall environmental aesthetics, and public security. Also, the direct effect of residential built environment on subjective well-being was proved with structural equation model.

Meanwhile, this study proved that subjective well-being has significant positive correlations with accessibility of destinations, overall environmental aesthetics, and public security. Good accessibility of destinations, which includes accessibility to sports venues, public facilities, parks, green land, etc., a beautiful environment, and high public security level could improve the residents’ subjective well-being. This result goes in line with those drawn by scholars in foreign academia. For example, Gao et al. proposed that short distance between destinations in daily travel encourages positive means of transportation and increases interpersonal interactions, which helps improve people’s mental health (Gao et al., 2016). Andrew and Dong et al. proposed that in a beautiful environment or a green area where there is more space and opportunities for physical activities and chances to interact with others, people can better improve their interpersonal relationships and mental health (Dannenberg et al., 2011). Frank et al. believe crimes are more likely to happen in an environment with poor public security. When people feel unsafe about the environment, they would bear more mental pressure and would not prefer walking or cycling trips (Frank et al., 2019). That’s because, in urban residential built environment, convenient facilities and high public security level are important premises for human interactions and connections. And green landscape inspires people’s curiosity and desire for exploration, which enhances residential well-being as well as social interactions and neighborhood unity.

No significant correlation was found between subjective well-being and the rest two built environment characteristics, namely proximity to supporting facilities and street connectivity. Though in some research, a high rate of land use could increase the residents’ subjective well-being as it makes the supporting facilities more convenient to use (Ma et al., 2020). Some others believed diversity has no significant relationship with subjective well-being. According to Cao, land used mix provides the residents with diversified destinations but simultaneously makes the community noisy and crowded, so its positive and negative impacts cancel each other out (Cao, 2016; Su et al., 2019). The situation is similar in the case of street connectivity. Although as noted by Leslie et al. research, higher street connectivity and crossroad density encourage walking trips and social interactions and improve mental health by creating a safe walking environment where cars drive much more slowly (Leslie and Cerin, 2008). But some research found street design may restrict residential mental health. For example, according to the research on low-income people in Southeast America by James et al., community walkability is positively correlated to depression, which is particularly true in poor areas. Because areas with high walkability are more likely to have problems like pollution and crimes, which may increase people’s mental pressure and thus impair mental health (James et al., 2017). In conclusion, subjective well-being could be influenced by multiple factors such as built environment, social environment, and individual factors. And for different built environment characteristics, their impacts may be multi-dimensional and contradictory. That explains why the research results of correlations between residential built environment characteristics and the residents’ subjective well-being vary from one another.

### Correlations between residential built environment, physical activities, and subjective well-being

The mediator model of this study found that, physical activities have a direct effect on subjective well-being and a mediating effect on the correlations between subjective well-being and residential built environment. Physical activity played a complete mediating role in Proximity to supporting facilities and Subjective well-being. Overall environmental aesthetics and Public security not only directly affect residents’ subjective well-being, but also have indirect effects on subjective well-being through physical activities. It may be because subjective well-being could be influenced by residential built environment through influence on commuting and individual health. This result goes in line with those drawn by scholars in foreign academia. For example, Craveiro and Erin et al. thought that with a higher accessibility of supporting facilities, and shorter commuting distance, the residents are more likely to choose green travel and thus have more physical activities, which in turn improve interpersonal interactions, mental health, and subjective well-being. People would commute more by walking when the community has various parks, squares, and green space, which improves health by creating more physical activities. People would also have enhanced subjective well-being after spending more time on physical activities and social interactions (Liu, 2016; Schmiedeberg and Schröder, 2017). In a beautiful environment or a green area where there is more space and opportunities for physical activities and chances to interact with others, people can better improve their interpersonal relationships and mental health (Dong and Qin, 2017). Meanwhile, a clear street network, safe traffic, and high public security level increase physical activities by encouraging the residents to commute by walking and enhance people’s physical and mental health as they feel safe and more comfortable when having sports activities.

Taken together, future planning on the construction of residential built environment in cities can be approached in the following aspects: (Lin and Yang, 2015) Under the backdrop of rapid urbanization, the residential built environment in cities shall try best to satisfy the needs of different social strata, especially the elderly, the female, the low-income, and the less educated groups. We shall maximize the aggregate of happiness by enhancing policy support for the improvement of old urban communities and the optimization of built environment that faces the whole society (Zhao et al., 2022). Supporting facilities for living and physical activities shall be easy to use and access with a diversified functional layout, so as to meet the various needs of the residents (Lu and Tan, 2015). Planning of activity venues in the residential area shall take full consideration of building practicability, environmental openness, and landscape aesthetics. Space and environment design are not only visual aesthetics for the residents, but also bear the responsibility to realize harmonious coexistence between man and nature. Outdoor built environment would be used more when people perceive a shorter distance to nature (Sallis, 2009). Street connectivity and security shall be improved with reasonable planning of public areas and outdoor activity space such as squares, parks, and green sidewalks. Measures shall be taken to reduce traffic flow and speed of motorized vehicles, so as to create a comfortable and safe environment for walking and cycling. It is also important to set up enough accessible facilities to meet the travelling needs of the vulnerable.

## Limitations and future directions

First, the study used a cross-sectional survey to explore the correlations between residential built environment, physical activities, and subjective well-being in cities of Yangtze River Delta. As it did not conduct longitudinal research, the results cannot explain the mutual influence between the variables. Second, this study used subjective measurements. While in future research, more objective research methods shall be considered to produce more objective results, such as GIS for the measure of built environment and accelerometer for physical activities. Third, in the next step, social environment and neighborhood relationship could be researched as another two influencing factors of physical activities and subjective well-being, so as to optimize the correlation model of residential built environment, physical activities, and subjective well-being.

## Conclusion

Lin and Yang (2015) residents with different individual characteristics may have different levels of perception and usage of built environment (Zhao et al., 2022). The intensity of physical activities has significant positive correlations with proximity to supporting facilities, accessibility of destinations, and public security (*p* < 0.05), while no significant correlation with overall environmental aesthetics and street connectivity (Lu and Tan, 2015). The residents’ subjective well-being has significant positive correlations with accessibility of destinations, overall environmental aesthetics, and public security (*p* < 0.05), while no significant correlation with proximity to supporting facilities and street connectivity (Sallis, 2009). Built environment characteristics have a direct effect on the residents’ physical activities and subjective well-being, with an effect value of 0.343 and 0.238, respectively. Physical activities not only have a direct effect on subjective well-being, with an effect value of 0.304, but also a mediating effect on the correlations between subjective well-being and built environment characteristics, which accounts for 30.42% of the total effect (Lu and Qin, 2013). Physical activity played a complete mediating role in Proximity to supporting facilities and Subjective well-being. Overall environmental aesthetics and Public security not only directly affect residents’ subjective well-being, but also have indirect effects on subjective well-being through physical activities. The indirect effect value accounted for 14.73 and 16.75% of the total effect value.

## Data availability statement

The raw data supporting the conclusions of this article will be made available by the authors, without undue reservation.

## Ethics statement

The studies involving human participants were reviewed and approved by Human Research Ethics Committee of Shanghai International Studies University. Written informed consent to participate in this study was provided by the participants, and if necessary, their legal guardian/next of kin.

## Author contributions

JZ designed the research and wrote the manuscript. YZ undertook the statistical analysis. MY and TW aided in reference collection and summary as well as participated in the manuscript preparation. QMF directed the research process and revised the draft. All authors contributed to the article and approved the submitted version.

## Conflict of interest

The authors declare that the research was conducted in the absence of any commercial or financial relationships that could be construed as a potential conflict of interest.

## Publisher’s note

All claims expressed in this article are solely those of the authors and do not necessarily represent those of their affiliated organizations, or those of the publisher, the editors and the reviewers. Any product that may be evaluated in this article, or claim that may be made by its manufacturer, is not guaranteed or endorsed by the publisher.
